# Supplementary material for: Identification of photoperiod-induced specific miRNAs in the adrenal glands of Sunite sheep (Ovis aries)
Source: Front Vet Sci. 2022 Jul 22;9:888207. doi: 10.3389/fvets.2022.888207 (PMC9354845; doi:10.3389/fvets.2022.888207)
Supplement: Supplementary file 1 [file Data_Sheet_1.ZIP › Supplementary file/miRDeep2 file.zip!/miRDeep2 file/Know_Structure/SPLP42A1/oar-mir-412.pdf]

miRBase precursor : oar-mir-412
Total read count : 474
oar-miR-412-3p read count : 459
oar-miR-412-5p read count : 7
remaining reads : 8

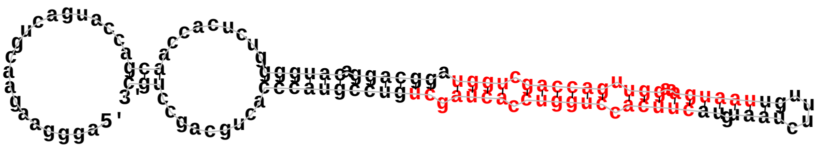

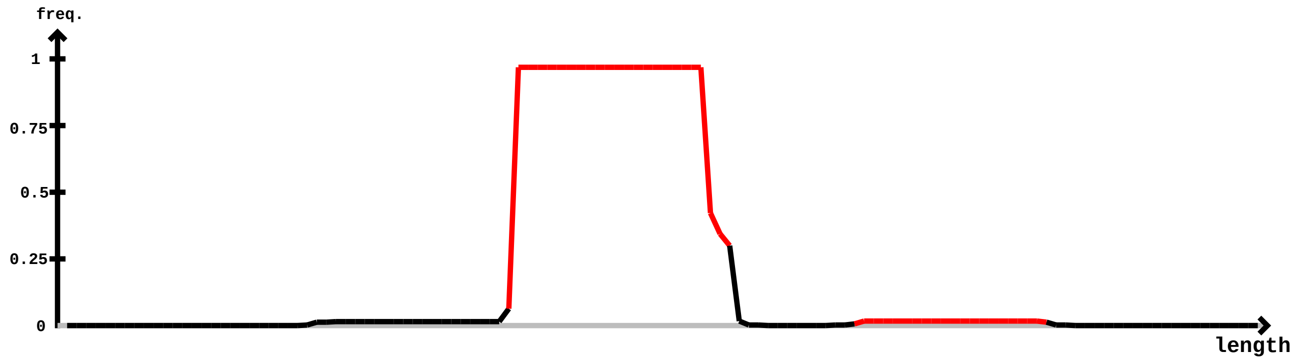

oar-miR-412-5p

oar-miR-412-3p

```
5'- agggaagaacgucaguaccagcaaccacucugggguacaggacggauggucgaccaguuggaaaguaauuguuucuaauguacuucaccugguccacuagcuguccguacccacugcagccugc   -3'    exp
    ..........(.((((..(((........)))((((((.((((((.((((.((((((.(((..((((((((....)))).))))))).)))))).)))).)))))))))))))))))).......   reads   mm
    ........................ccacucugggguacaggacgga..............................................................................   1       0
    .........................cacucugggguacaggacgg...............................................................................   1       0
    .........................cacucugggguacaggacgga..............................................................................   4       0
    ...........................cucugggguacaggacgga..............................................................................   1       0
    .............................................auggucgaccaguuggaaagu..........................................................   24      0
    ..............................................uggucgaccaguuggaaagu..........................................................   235     0
    ..............................................uggucgaccaguuggaaagua.........................................................   37      0
    ..............................................uggucgaccaguuggaaaguaa........................................................   21      0
    ..............................................uggucgaccaguuggaaaguaau.......................................................   134     0
    ..............................................uggucgaccaguuggaaaguaauu......................................................   7       0
    ..............................................uggucgaccaguuggaaaguaauugu....................................................   1       0
    ...............................................................................guacuucaccugguccacuagcu......................   1       0
    .................................................................................acuucaccugguccacuagc.......................   1       0
    .................................................................................acuucaccugguccacuagcu......................   1       0
    ..................................................................................cuucaccugguccacuagc.......................   1       0
    ..................................................................................cuucaccugguccacuagcu......................   3       0
    ..................................................................................cuucaccugguccacuagcugu....................   1       0
```
